# Intracavitary cardiac lipoma: a benign tumour requiring a complex treatment: a case report

**DOI:** 10.1093/ehjcr/ytaf436

**Published:** 2025-09-05

**Authors:** Michalina Mazurkiewicz, Dominika Blachut, Tomasz Hrapkowicz, Jerzy Nożyński, Tomasz Kukulski

**Affiliations:** 2nd Chair of Cardiology, Faculty of Medical Sciences in Zabrze, Medical University of Silesia, 41-800 Zabrze ul C.Sklodowskiej 10, Poland; Department of Clinical Cardiology Specialistic Hospital in Zabrze, 41-800 Zabrze ul.C.Sklodowskiej 9, Poland; 2nd Chair of Cardiology, Faculty of Medical Sciences in Zabrze, Medical University of Silesia, 41-800 Zabrze ul C.Sklodowskiej 10, Poland; Department of Clinical Cardiology Specialistic Hospital in Zabrze, 41-800 Zabrze ul.C.Sklodowskiej 9, Poland; Department of Cardiac Surgery, Heart and Lung Transplantation and Mechanical Circulatory Support, Silesian Center for Heart Diseases, Zabrze 41-800, ul.C.Sklodowskiej 10, Poland; Histopathology Laboratory, Silesian Center for Heart Diseases, Zabrze 41-800, ul.CSklodowskiej 10, Poland; 2nd Chair of Cardiology, Faculty of Medical Sciences in Zabrze, Medical University of Silesia, 41-800 Zabrze ul C.Sklodowskiej 10, Poland; Department of Clinical Cardiology Specialistic Hospital in Zabrze, 41-800 Zabrze ul.C.Sklodowskiej 9, Poland

**Keywords:** Echocardiography, Cardiac lipoma, Cardiosurgery, Neoplasms, Tumour, Case report

## Abstract

**Background:**

Primary cardiac tumours are rare, and cardiac lipomas represent only 8.4% of these cases. Cardiac lipomas consist of two pathologically distinct types: lipomas, and lipomatous hypertrophy of the interatrial septum (LHIS). Both types can be challenging to differentiate through imaging and are often reported together in the literature, complicating an accurate count of each type. Cardiac lipomas can occur in all chambers of the heart, most commonly in the atrial septum and the right atrium, often presenting incidentally or during evaluation of non-specific symptoms.

**Case summary:**

This report presents a case of a 54-year-old female, who was incidentally found to have a large cardiac tumour during a routine abdominal ultrasound. Transthoracic and transoesophageal echocardiography revealed a 65 × 58 × 55 mm tumour occupying the right atrium. Initial suspicion pointed to a myxoma; however, surgical and pathological analysis confirmed the diagnosis of cardiac lipoma. The patient underwent complex cardiac surgery, which included tumour resection, atrial wall reconstruction, PFO closure, tricuspid ring implantation and left anterior descending artery (LAD) grafting using left internal mammary artery (LIMA). The patient recovered well however due to the perioperative complete atrioventricular (AV) block required permanent pacemaker implantation. The patient remains under regular echocardiographic follow-up to monitor for potential recurrence.

**Discussion:**

This case underscores the importance of surgical intervention for benign large intracavitary tumours to prevent mechanical and haemodynamic complications and improve long-term outcome. Carefully planning of surgical treatment should take into account the complexity of the procedure and potential conduction disturbances.

Learning points

- Even the absence of specific symptoms does not rule out the presence of an expansive tumour in the heart.
- Surgical treatment of cardiac tumours can involve a number of complex additional invasive procedures, which should be considered when qualifying a patient for surgery.

## Introduction

Primary cardiac tumours are rare, with an overall incidence ranging from od 0001% to 0,3%. Among benign tumours, cardiac lipomas account for only 8.4% of primary cardiac tumours.^1^ These lesions include two distinct pathologically defined entities: Lipoma and lipomatous hypertrophy of the interatrial septum (LHIS). These entities differ in their pathomorphological cellular distribution. A lipoma is a homogeneous, encapsulated collection of mature adipocytes with minimal cellular atypia. In contrast, LHIS represents an overgrowth of adipocytes within normal heart tissue, making differentiation on imaging studies challenging. Surgical literature often groups these entities together, complicating the determination of the actual incidence of each pathological entity in most reports.^2^

These lesions can be found in all heart chambers, with the atrial septum being the most common site (39%)^3,4^—mainly in cases of LHIS—followed by the right atrium (RA) (18%),^5,6^ left ventricle (LV) (12%),^7^ and right ventricle (RV) (5%).^8^ They originate from the subendocardium (50%), subepicardium (25%), or myocardium (25%) and vary in size.

We present a clinical case of a patient in whom a cardiac tumour, incidentally detected, turned out to be a highly expansive lipoma growing from the wall of the right atrium.

## Summary figure

**Figure:**
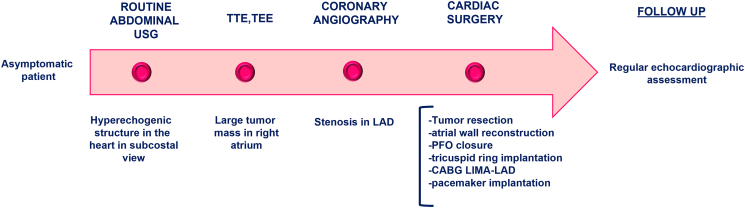


## Case report

**Figure 1:**
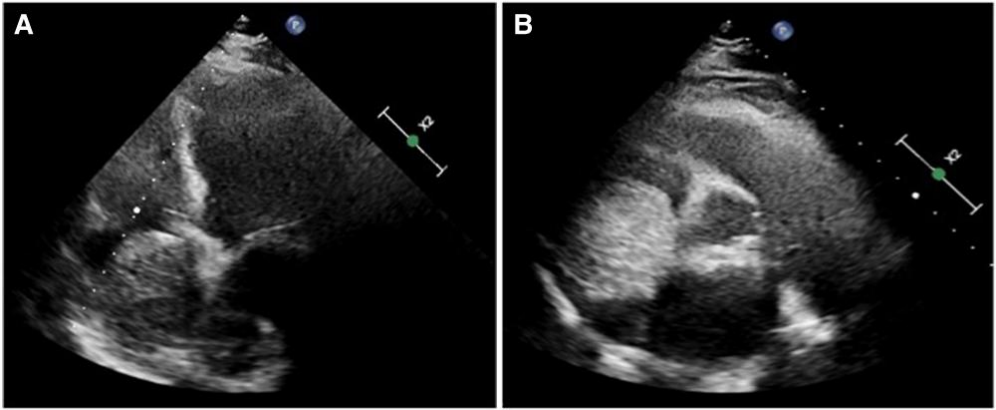
Transthoracic echocardiography—tumour in right atrium. (*A*) Apical four-chamber view; (*B*) parasternal short axis view at the aortic level). Own study.

**Figure 2:**
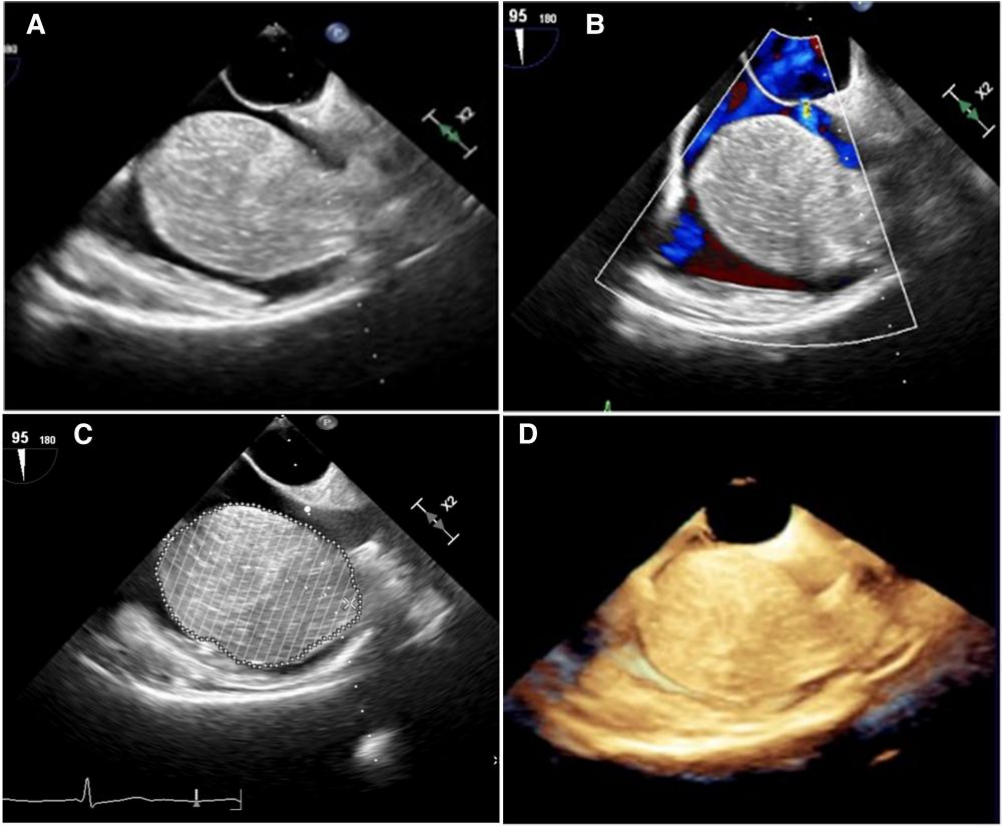
Transoesophageal echocardiography. (*A*) Combined bicaval view. (*B*) Combined bicaval view depicting opened PFO interatrial shunt, (*C*) measurement of tumour volume by Simpson method—136 mL, (*D*) 3D visualization. Own study.

A 54-year-old female patient with a medical history of arterial hypertension and hypothyroidism, currently in a state of euthyroidism, was referred to department with a preliminary diagnosis of right atrial myxoma, incidentally detected during a routine abdominal ultrasound examination. Further assessment with transthoracic echocardiography revealed a large tumour mass occupying nearly the entire right atrium. (*Figure 1A* and *B*) Transoesophageal echocardiography provided a detailed evaluation of the solid oval structure, showing a lobular structure attached to the right atrial vault by a wide (23 mm) pedicle. The tumour was slightly mobile and did not interfere with the flow through the tricuspid valve (see the subsequent Videos 1 and 2). Its size was measured with maximal diameters of 65 × 58 × 55 mm, a calculated area of 35 cm², and a volume of 136 mL using Simpson’s method. Additionally, a patent foramen ovale (PFO) with a spontaneous, haemodynamically insignificant left-to-right shunt was detected, (*Figure 2A–D*), left ventricular ejection fraction was normal. An additional routine imaging test performed upon admission to our department was a chest X-ray, which revealed a slightly prominent right heart border.

**Figure 3:**
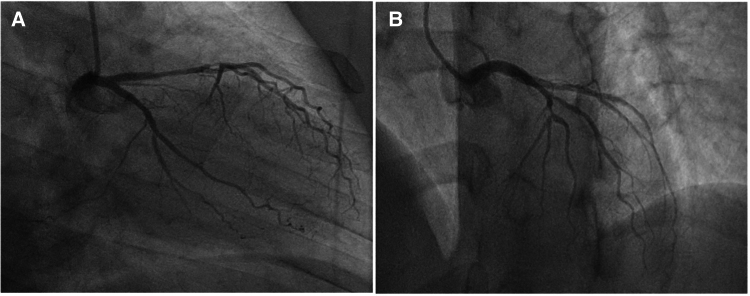
Coronary angiography. (*A*) RAO 40, caudal view: significant LAD stenosis in segment 7; (*B*) AP 0, cranial view: significant stenosis in segment 7. Own study.

As part of preoperative evaluation, the patient also underwent coronary angiography, which revealed significant stenosis in the distal left anterior descending artery (LAD) (*Figure 3A* and *B*). Physical examination and laboratory tests showed no significant abnormalities. The C-reactive protein level was 1.21 mg/L (normal range: 0–5 mg/L), N-terminal pro-B-type natriuretic peptide (NT-proBNP) was 255.1 pg/mL (normal range: 0–125 pg/mL), and serum troponin T level was 16.12 ng/L (normal range: 0–14 ng/L).

During cardiac surgery, after cardioplegia, a tumour was found grossly extending into the roof and lateral wall of the right atrium, reaching the interatrial septum. Following tumour resection, along with part of the atrial wall, the dilated tricuspid annulus was plicated and stabilized using E-L MC3 Tricuspid Ring 32 mm, and the PFO was closed. The right atrium was reconstructed using bovine pericardium.

**Figure 4:**
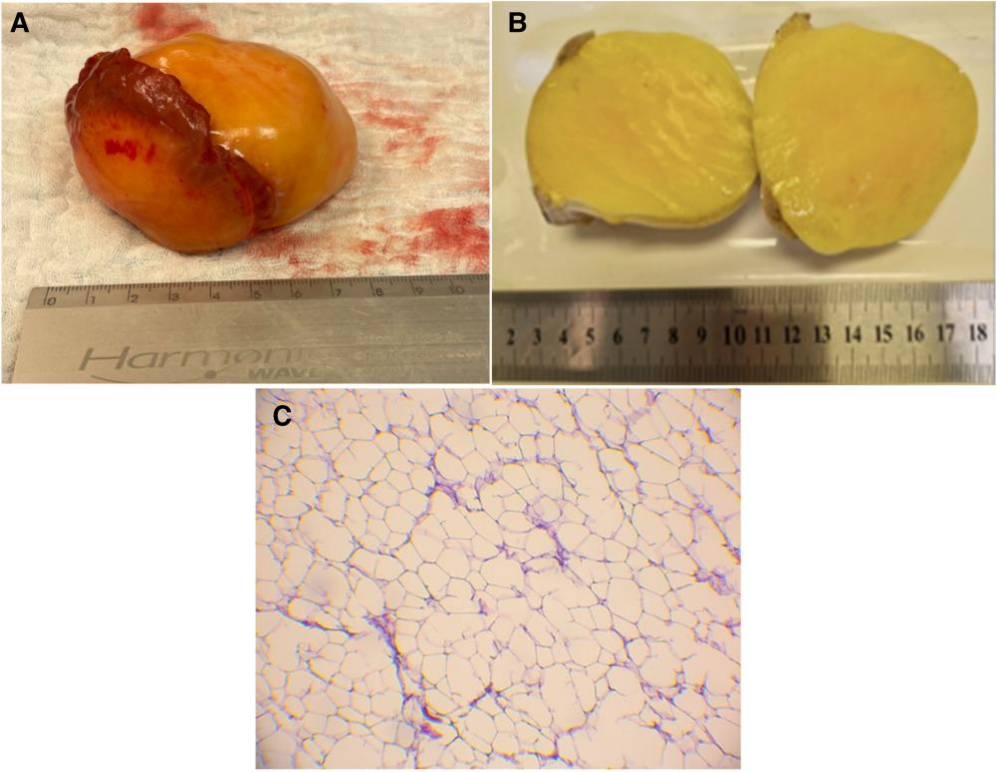
(*A*) Dissection preparation. (*B*) Macroscopic cross-sectional assessment of the tumour. (*C*) Lipoma—microscopical image of resected tumour with yellow adipose tissue cells. Magnification 100-fold. H&E staining. Own study.

The final step of the procedure involved sewing a left internal mammary artery (LIMA) bypass graft into the LAD. In gross inspection, the tumour presented an almost certain diagnosis of lipoma. Postoperative macroscopic and microscopic analysis confirmed the diagnosis of cardiac lipoma (*Figure 4A–C*).

**Figure 5:**
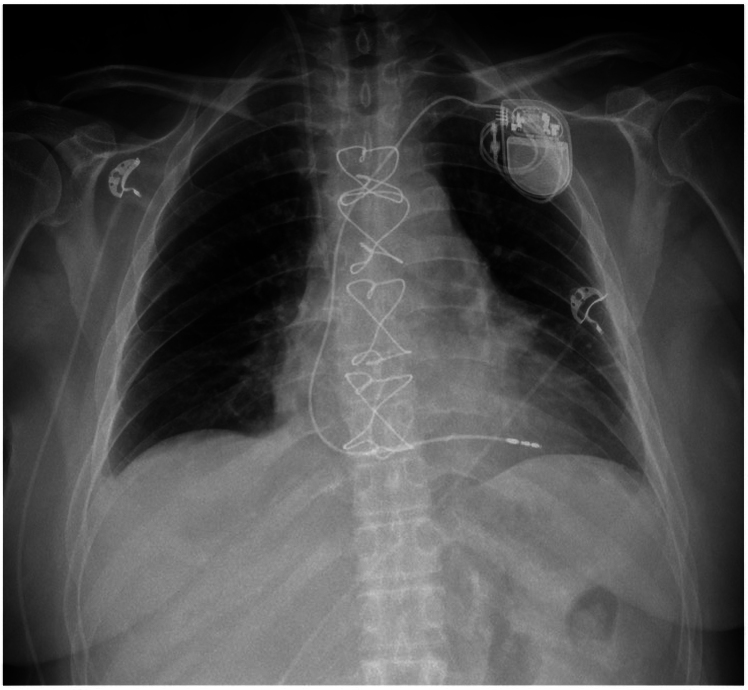
Anterior posterior (AP) chest X-ray confirmed the pacemaker and lead position. Own study.

During the procedure, a complete atrioventricular block occurred. Initially, the patient was temporarily protected with an endocavitary electrode. The conduction disturbance proved irreversible and was caused by extensive resection of the right atrium. Ultimately, the patient required permanent pacemaker implantation (*Figure 5*).

The patient remains under regular echocardiographic surveillance as well as pacemaker monitoring. A follow-up echocardiography after the procedure revealed no signs of recurrence (no additional echogenic structures in the right atrium and demonstrated good tricuspid valve function).

## Discussion

The diagnostic workup of cardiac tumours typically involves imaging studies; however, such tumours are most often discovered incidentally or during investigation of non-specific symptoms. Cases of dyspnoea or palpitations are reported, although the majority remain asymptomatic,^9^ as in this case. The gold standard for diagnosing cardiac tumours is transthoracic and transoesophageal echocardiography. Additional imaging studies, such as CT and MRI, are complementary diagnostic methods that are useful when the diagnosis is uncertain. They allow a more accurate determination of the size and location of the tumour and are helpful in structure assessment.^9–11^ In the present case, additional imaging studies were withheld due to the high echocardiographic likelihood of a right atrial myxoma. Furthermore, the considerable size of the mass raised significant concern, and the patient was therefore urgently referred for surgical treatment.

In cases of cardiac tumours, particularly those of significant size, surgical intervention is the optimal therapeutic approach to prevent mechanical and haemodynamic complications caused by tumour growth.^12^

The initially planned goals of surgical treatment (tumour resection and LIMA–LAD bypass) were exceeded in this case. The procedure was extended to include tricuspid valve repair with an annuloplasty ring, closure of a PFO, and a significantly more extensive resection of the right atrium than originally anticipated, which resulted in permanent atrioventricular conduction impairment.

Cardiac tumours can reach very large sizes and may be located in any chamber of the heart. Rainer *et al*. advanced the ‘invasion hypothesis,’ suggesting that an invasive cardiac lipoma in the right atrium of the heart may originate in the subendocardium. As the tumour grows, the pressure exerted by the adipose tissue leads to stretching of the atrial wall, which leads to collapse of the tumour mass and replacement of the atrial wall.^5,7,13^

The limitations in current knowledge are mainly due to the small number of cases and the lack of large clinical trials. Information on cardiac lipomas comes mainly from case reports. Continuing research in this area aims to identify genetic, metabolic, and environmental factors that may be responsible for the development of lipomas, and to develop new therapeutic approaches that would benefit patient treatment and care.

## Conclusions

This case report describes a rare cardiac tumour in the form of a highly expansive lipoma. The patient underwent extensive cardiac surgical procedures with very good outcomes. She remains under regular echocardiographic follow-up to monitor for recurrence.

## Lead author biography


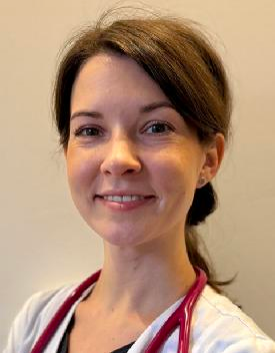


Michalina Mazurkiewicz is a cardiology resident at the Department of Cardiology in the Specialistic Hospital in Zabrze, Poland. She graduated from the Medical University of Silesia in 2020. Her primary interests include echocardiography, heart failure, and valvular heart disease.

**Consent:** The authors confirm that written consent for the submission and publication of this case report, including images and associated text, has been obtained from the patient in accordance with COPE guidance.

## Data Availability

The data that support the findings of this study comes directly from in-hospital medical records and as such are not available in a public domain. However some of the data i.e. additional imaging data could be available from the corresponding author [T.K.], upon reasonable request.
